# Supplementary material for: A subpopulation of cortical VIP-expressing interneurons with highly dynamic spines
Source: Commun Biol. 2022 Apr 13;5:352. doi: 10.1038/s42003-022-03278-z (PMC9008030; doi:10.1038/s42003-022-03278-z)
Supplement: Supplementary file 2 — Supplementary Information [file 42003_2022_3278_MOESM2_ESM.pdf]

Supplementary Figures to manuscript:

A subpopulation of cortical VIP-expressing interneurons with highly dynamic spines

Christina Georgiou, Vassilis Kehayas, Kok Sin Lee, Federico Brandalise, Daniela A. Sahlender, Jerome Blanc, Graham Knott, Anthony Holtmaat

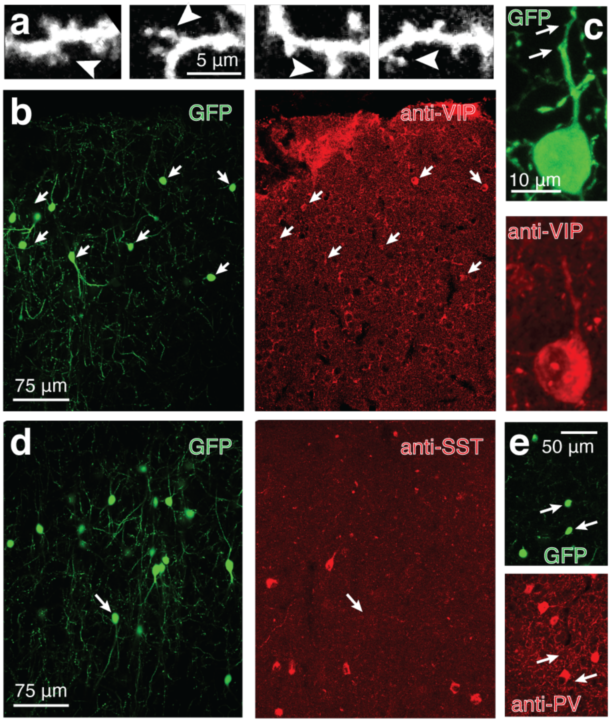

Supplementary Figure 1.

(a) Examples of spiny dendrites in the cortex of VIP-IRES-Cre mice crossbred with the Ai14 reporter line. Arrowheads point to examples of spines. (b-e) Immunolabeling of VIP, SST and PV in sections of VIP-IRES-Cre mice transfected with AAV-pCAG-FLEX-EGFP-WPRE. (b) Examples of GFP-expressing cells (green) that are co-labeled with anti-VIP antibodies (red, arrowheads). (c) High-magnification image of a GFP-positive neuron (green) that is co-labeled with anti-VIP antibodies (red). Arrows point to dendritic protrusions that resemble spines. (d, e) Examples of GFP-expressing neurons (green) that are devoid of anti-SST (d) and anti-PV staining (e).

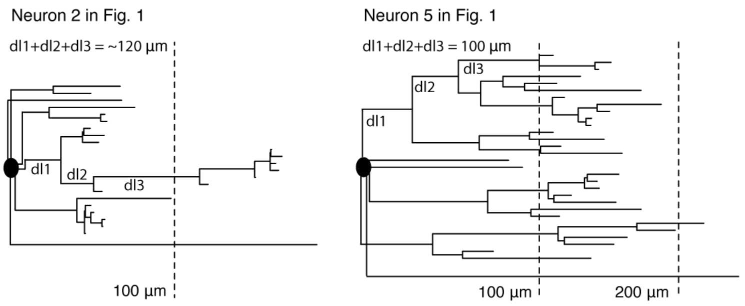

Supplementary Figure 2.

Dendrograms of neuron 2 and 5 in Figure 1, showing the two main types of branching patterns.

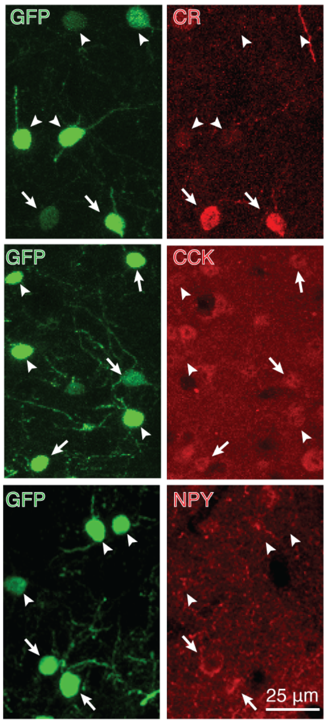

Supplementary Figure 3.

Examples of GFP-expressing neurons that are co-labeled with anti-CR, anti-CCK, and anti-NPY antibodies (arrow heads) or negative for either marker (arrows).

a

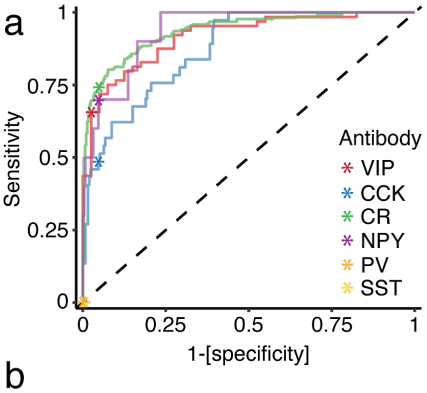

b

AB	Sensitivity	1-Specificity
VIP	0.66	0.03
CR	0.49	0.05
NPY	0.75	0.05
CCK	0.70	0.05
PV	0.00	0.00
SST	0.00	0.00

Supplementary Figure 4.

(a) ROC curve of the GLM's classification performance with varying thresholds on the probability estimates for the various antibody labeling. (b) For each antibody (AB), the sensitivity and 1-specificity metrics are reported, for the highest value of 1-specificity lower than 0.05.

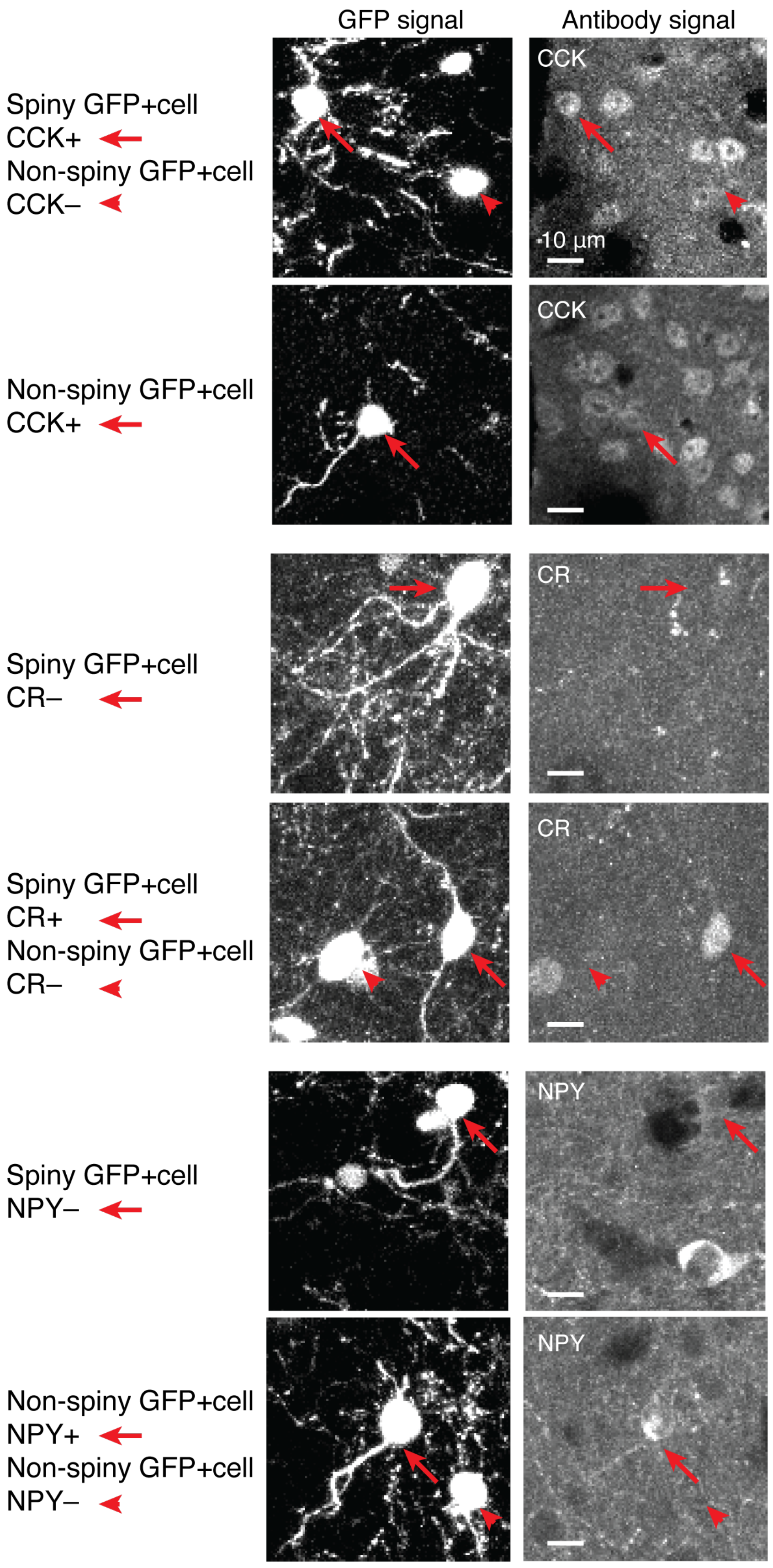

Supplementary Figure 5.

Examples of spiny and non-spiny GFP-expressing cells, co-labeled or negative for CCK, CR and NPY.

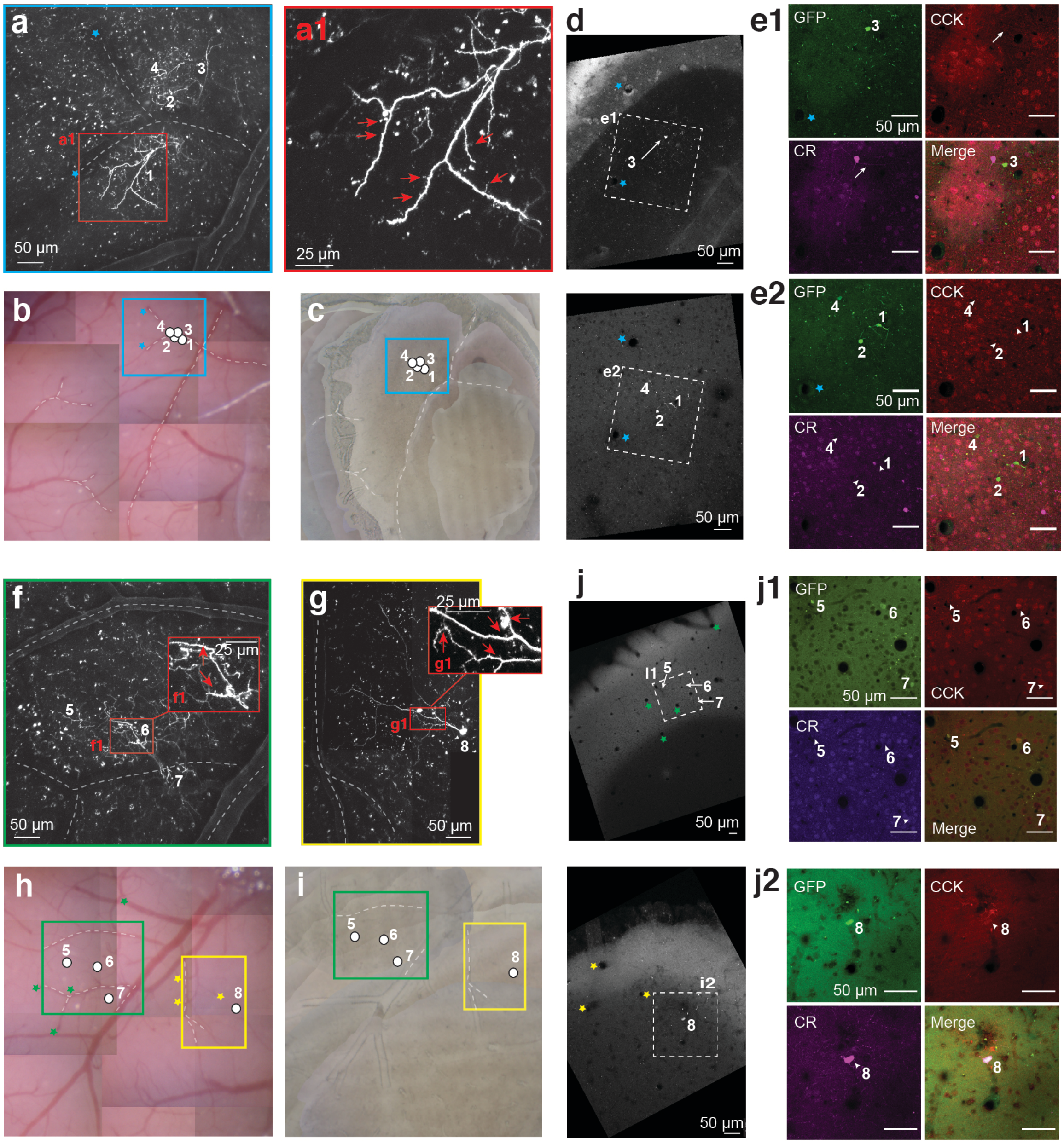

k

Cell	Spiny	CCK	CR	Panel
1	+	–	–	e2
2	+	–	–	e2
3	–	–	–	e1
4	–	–	–	e2
5	–	+	–	j1
6	+	+	–	j1
7	–	–	–	j1
8	+	–	+	j2
9	–	–	–	ns
10	–	–	–	ns
11	–	–	–	ns

Supplementary Figure 6.

Correlative in vivo imaging and immunofluorescence microscopy. (a, f and g) GFP-expressing dendrites (AAV-DIO-GFP in VIP-cre mice) imaged in vivo through a cranial window. Some of the blood vessels that run over the surface are indicated with dotted lines. The points at which they plunge into

the parenchyma are indicated with asterisks. (a1, f1 and g1) high magnifications of GFP-dendrites with spines (arrows). (b and h) bright field images of the vasculature on the brain's surface through the cranial windows. The red, green and yellow boxes refer to the positions of the imaged regions in a, f and g. The matching vasculature with the in vivo fluorescence images is indicated by dotted lines and asterisks. The numbered dots indicate the approximate positions of the neurons that were imaged in vivo. (c and i) stacks of perfusion-fixed brain sections with the matching vasculature (dotted lines, asterisks) and positions of the imaged regions (boxes) and neurons (numbered dots). (d and j) confocal images of the cell bodies that belong to the in vivo-imaged dendrites, and the vasculature fiducial points (asterisks). (e1, e2, j1 and j2) immunofluorescence confocal images of the regions with the GFP-expressing cell bodies (indicated by the dotted boxes in d and j). Cell bodies were variably immunopositive or negative for CCK and CR. (k) Summary of the various combinations of immunostaining. Cell 1 and 2 were spiny but negative for either CCK and CR. Cell 6 was spiny and positive for CCK but negative for CR. Conversely, cell 8 was spiny and negative for CCK but positive for CR. The other cells were not convincingly spiny. Spine 9-11 are not shown (ns).

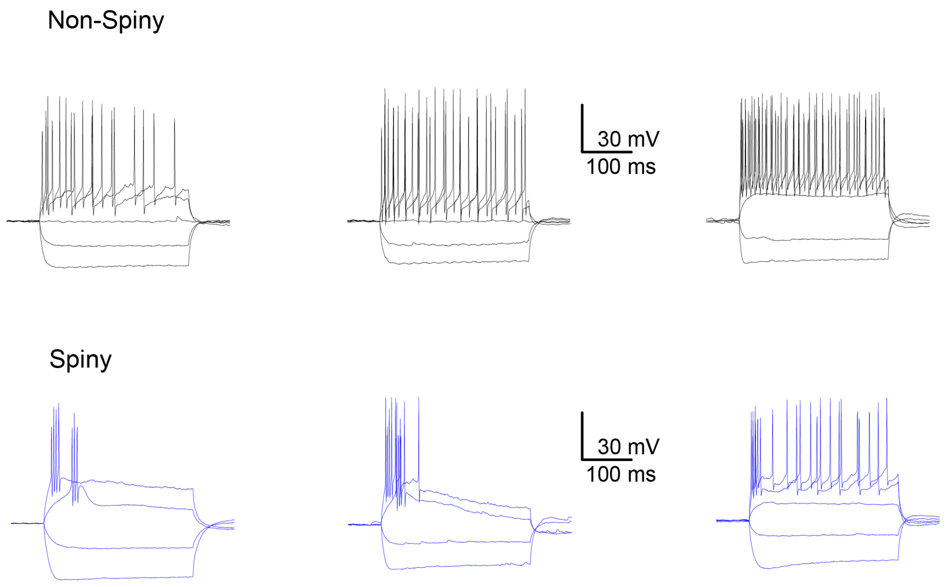

Supplementary Figure 7.

Examples of spiking patterns of non-spiny vs spiny neurons. Spiny neurons are characterized by stronger burst spiking properties.

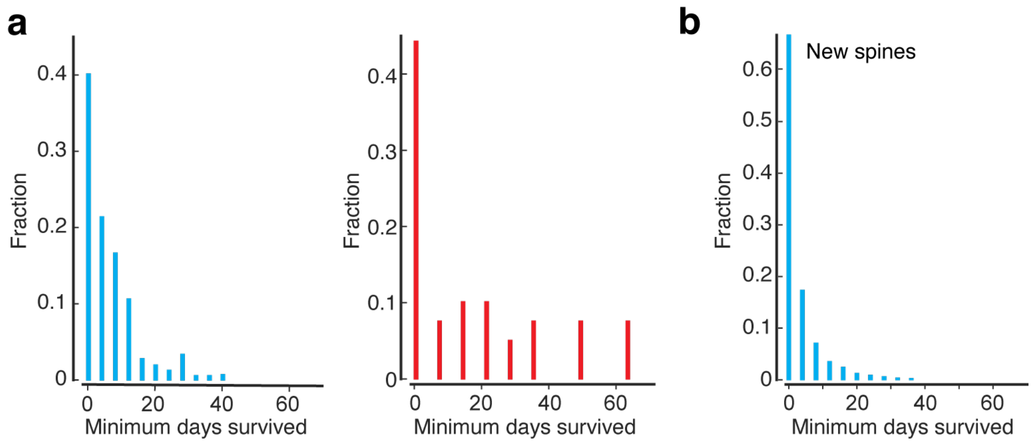

Supplementary Figure 8.

(a) Relative probability of survival of spines present in the first imaging session for VIP (left, blue) and pyramidal neurons (right, red). (b) Relative probability of new spine survival for VIP neurons.

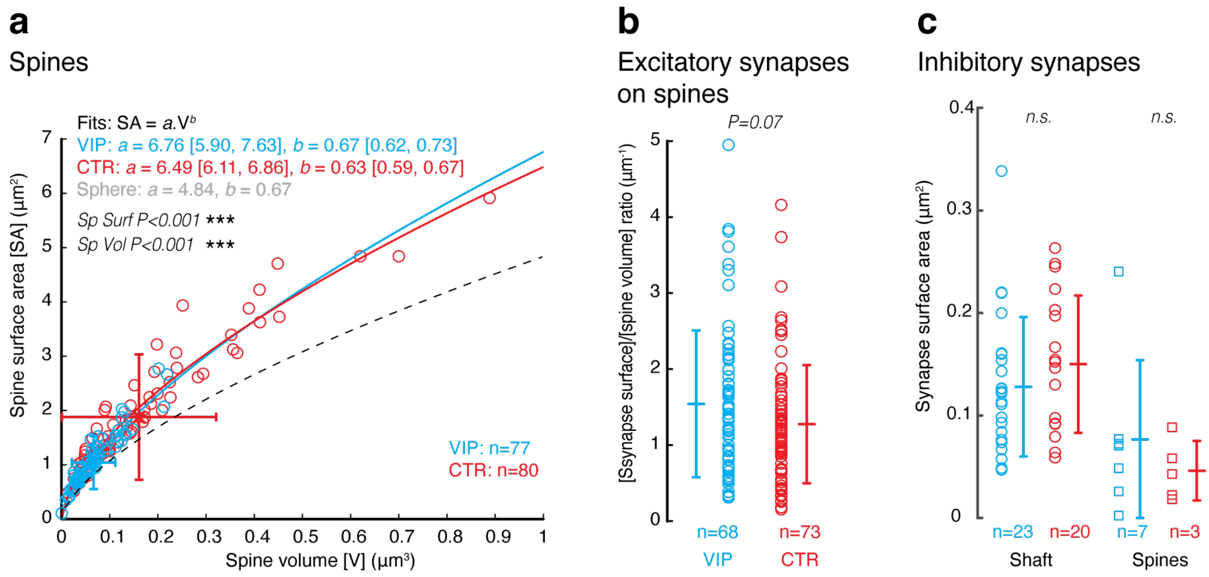

Supplementary Figure 9.

(a) Relationship between spine volumes and spine surface area for VIP and CTR dendrites with averages ± s.d., as quantified using SBEM. The lines indicate non-linear fits, suggesting that VIP and CTR spines have similar volume to surface relationships. The dotted line indicates the relationship for perfect spheres. (b) The surface area-to-spine volume ratio is not significantly different for VIP spines as compared to CTR spines. (c) The inhibitory synapse surface area on dendritic shafts and spines is not different between VIP and CTR dendrites.
