# Supplementary material for: A subpopulation of cortical VIP-expressing interneurons with highly dynamic spines
Source: Commun Biol. 2022 Apr 13;5:352. doi: 10.1038/s42003-022-03278-z (PMC9008030; doi:10.1038/s42003-022-03278-z)
Supplement: Supplementary file 3 — Reporting Summary [file 42003_2022_3278_MOESM3_ESM.pdf]

nature portfolio

Corresponding author(s): Anthony Holtmaat

Last updated by author(s): Sep 30, 2021

Reporting Summary

Nature Portfolio wishes to improve the reproducibility of the work that we publish. This form provides structure for consistency and transparency in reporting. For further information on Nature Portfolio policies, see our Editorial Policies and the Editorial Policy Checklist.

Statistics

For all statistical analyses, confirm that the following items are present in the figure legend, table legend, main text, or Methods section.

n/a	Confirmed	
☐	☒	The exact sample size (n) for each experimental group/condition, given as a discrete number and unit of measurement
☐	☒	A statement on whether measurements were taken from distinct samples or whether the same sample was measured repeatedly
☐	☒	The statistical test(s) used AND whether they are one- or two-sided<br>*Only common tests should be described solely by name; describe more complex techniques in the Methods section.*
☐	☒	A description of all covariates tested
☒	☐	A description of any assumptions or corrections, such as tests of normality and adjustment for multiple comparisons
☐	☒	A full description of the statistical parameters including central tendency (e.g. means) or other basic estimates (e.g. regression coefficient) AND variation (e.g. standard deviation) or associated estimates of uncertainty (e.g. confidence intervals)
☐	☒	For null hypothesis testing, the test statistic (e.g. F, t, r) with confidence intervals, effect sizes, degrees of freedom and P value noted<br>*Give P values as exact values whenever suitable.*
☒	☐	For Bayesian analysis, information on the choice of priors and Markov chain Monte Carlo settings
☐	☒	For hierarchical and complex designs, identification of the appropriate level for tests and full reporting of outcomes
☒	☐	Estimates of effect sizes (e.g. Cohen's d, Pearson's r), indicating how they were calculated

Our web collection on statistics for biologists contains articles on many of the points above.

Software and code

Policy information about availability of computer code

Data collection	Scanimage was used to image neuronal structure (http://www.scanimage.org) running on MATLAB 2017a.<br>Electrophysiology data were acquired using pClamp command/record software (Axon Instruments)<br>Elelectron microscope images were acquired using commercial software of the 3View system (Gatan with Zeiss microscope) or the focused ion beam scanning electron microscope (NVision 40, Zeiss NTS).
Data analysis	Custom-written scripts in MATLAB (version 2017a, Mathworks) and custom-written ImageJ (version 2.0.0; http://rsbweb.nih.gov/ij/) plugins were used to process the 2-photon images and analyze imaging data.

For manuscripts utilizing custom algorithms or software that are central to the research but not yet described in published literature, software must be made available to editors and reviewers. We strongly encourage code deposition in a community repository (e.g. GitHub). See the Nature Portfolio guidelines for submitting code & software for further information.

Data

Policy information about availability of data

All manuscripts must include a data availability statement. This statement should provide the following information, where applicable:

- Accession codes, unique identifiers, or web links for publicly available datasets
- A description of any restrictions on data availability
- For clinical datasets or third party data, please ensure that the statement adheres to our policy

Upon publication of the manuscript, the data used to generate the figures will be made freely available at the CERN data repository Zenodo https://zenodo.org/communities/holtmaat-lab-data. The principal Matlab code that was used for data analysis will also be freely available at the CERN data repository and Github. We have reserved a DOI for this repository: 10.5281/zenodo.6137441.

Field-specific reporting

Please select the one below that is the best fit for your research. If you are not sure, read the appropriate sections before making your selection.

☒ Life sciences ☐ Behavioural & social sciences ☐ Ecological, evolutionary & environmental sciences

For a reference copy of the document with all sections, see nature.com/documents/nr-reporting-summary-flat.pdf

Life sciences study design

All studies must disclose on these points even when the disclosure is negative.

Sample size	We did not perform sample size calculations. Sample sizes were based on previous and similar types of studies (For example Holtmaat et al. 2006; Schubert et al. 2013; Cane et al. 2014). These sample sizes are considered adequate and consistent with the existing literature.
Data exclusions	All acquired data were included for analysis, and will be available in the data repository. None were excluded from the graphs.
Replication	Longitudinal in vivo imaging was followed by immunocytochemistry or electron microscopy. Analyzed parameters were different from the in vivo imaging. Electrophysiology was performed on a separate set of mice. For figure 4 data were reused from Schubert et al. 2013. This is stated in the manuscript.
Randomization	For the main data set, 4-6 week-old male mice were acquired from a commercial provider, and all received the same surgical procedures except for some immunocytochemistry and electrophysiology experiments.
Blinding	The operators were not be blinded to any conditions, since all experiments were performed in longitudinal fashion and did not concern group comparisons. For electrophysiology and EM, cells and dendrites needed to be a priori selected by the experimenter for efficient analysis.

Reporting for specific materials, systems and methods

We require information from authors about some types of materials, experimental systems and methods used in many studies. Here, indicate whether each material, system or method listed is relevant to your study. If you are not sure if a list item applies to your research, read the appropriate section before selecting a response.

Materials & experimental systems

n/a	Involved in the study	
☐	☒	Antibodies
☒	☐	Eukaryotic cell lines
☒	☐	Palaeontology and archaeology
☐	☒	Animals and other organisms
☒	☐	Human research participants
☒	☐	Clinical data
☒	☐	Dual use research of concern

Methods

n/a	Involved in the study	
☒	☐	ChIP-seq
☒	☐	Flow cytometry
☒	☐	MRI-based neuroimaging

Antibodies

Antibodies used	The following antibodies were used: goat anti-parvalbumin (Swant, PVG-214), rabbit anti-vasoactive intestinal polypeptide (Immunostar, #20077), rat anti-somatostatin (MerckMillipore, MAB354), goat anti-calretinin (Swant, CG1), rabbit anti-neuropeptide Y (Abcam, ab10980), and rabbit anti-cholecystokinin (Sigma, SAB2100357). As secondary antibodies, we used alexa 568/647 anti-rabbit, alexa 568 anti-rat, and alexa 568 anti-goat (Thermofisher).
Validation	Validation data from providers.<br>VIP antibody (Immunostar): The specificity of the antiserum was examined by soluble pre-adsorption with the peptides in question at a final concentration of 10-5 M, at which VIP immunolabeling was completely abolished. Pre-adsorption with many other peptides resulted in no reduction of immunostaining.<br>PV antibody (Swant): Antiserum PVG 213 does not stain the brain of parvalbumin knock out mice<br>SST antibody (Merck): Recognizes Somatostatin. Shows no cross-reactivity to enkephalins, other endorphins, substance P or CGRP. Partially cross-reacts with somatostatin fragments.<br>CR antibody (Swant): Antibody CG1 does not stain the brain of calretinin knock out mice<br>NPY antibody (Abcam): no data available<br>CCK antibody (Sigma): verified on Western blot, not on sections.

Animals and other organisms

Policy information about studies involving animals; ARRIVE guidelines recommended for reporting animal research

Laboratory animals	C57Bl/6J wild-type (males and females). Surgeries (start of the experiment) were done on 4-6 weeks old mice and imaging experiments finished at 12-15 weeks-old. Animals were housed under standardized conditions in the animal facilities of the University of Geneva.
Wild animals	This study did not involve the use of wild animals.
Field-collected samples	This study did not involve the use of field collected samples.
Ethics oversight	All experiments were performed in accordance with the guidelines of the Swiss Federal Act on Animal Protection and Swiss Animal Protection Ordinance. The ethics committee of the University of Geneva and the Cantonal Veterinary Office (Geneva, Switzerland) approved all experiments (licenses GE/28/14, GE/61/17, and GE/121/19).

Note that full information on the approval of the study protocol must also be provided in the manuscript.
